# Aquaporin-8 transports hydrogen peroxide to regulate granulosa cell autophagy

**DOI:** 10.3389/fcell.2022.897666

**Published:** 2022-08-23

**Authors:** Binbin Huang, Lingling Jin, Luodan Zhang, Xiaolin Cui, Zhen Zhang, Yongqi Lu, Lujia Yu, Tonghui Ma, He Zhang

**Affiliations:** ^1^ Department of Pathophysiology, College of Basic Medical Sciences, Dalian Medical University, Dalian, China; ^2^ Department of Maternal, Child and Adolescent Health, School of Public Health, Anhui Medical University, MOE Key Laboratory of Population Health Across Life Cycle, NHC Key Laboratory of Study on Abnormal Gametes and Reproductive Tract, Anhui Provincial Key Laboratory of Population Health and Aristogenics, Hefei, Anhui, China; ^3^ Department of Nephrology, Anhui Provincial Children’s Hospital, Hefei, Anhui, China

**Keywords:** AQP8, hydrogen peroxide, granulosa cell, autophagy, follicular atresia

## Abstract

Aquaporin-8 (AQP8), a member of the aquaporin family, is strongly expressed in follicular granulosa cells, which could affect the hormone secretion level in females. AQP8, as a membrane protein, could mediate H_2_O_2_ into cells, thereby triggering various biological events. The deficiency of *Aqp8* increases female fertility, resulting from the decrease in follicular atresia. The low cell death rate is related to the apoptosis of granulosa cells. However, the mechanism by which AQP8 regulates the autophagy of granulosa cells remains unclear. Thus, this study aimed to explore the effect of AQP8 on autophagy in follicular atresia. We found that the expression of the autophagy marker light-chain protein 3 was significantly downregulated in the granulosa cells of *Aqp8*-knockout (*Aqp8* ^*−/−*^) mice, compared with wild-type (*Aqp8* ^*+/+*^) mice. Immunofluorescence staining and transmission electron microscopic examination indicated that the number of autophagosomes in the granulosa cells of *Aqp8* ^*−/−*^ mice decreased. Using a follicular granulosa cell autophagy model, namely a follicular atresia model, we verified that the concentration of H_2_O_2_ significantly increased during the autophagy of granulosa cells, consistent with the *Aqp8* mRNA level. Intracellular H_2_O_2_ accumulation was modulated by endogenous AQP8 expression level, indicating that AQP8-mediated H_2_O_2_ was involved in the autophagy of granulosa cells. AQP8 deficiency impaired the elevation of H_2_O_2_ concentration through phosphorylated tyrosine activation. In addition, we carried out the analysis of transcriptome sequencing datasets in the ovary and found there were obvious differences in principal components, differentially expressed genes (DEGs) and KEGG pathways, which might be involved in AQP8-regulated follicular atresia. Taken together, these findings indicated that AQP8-mediated H_2_O_2_ transport could mediate the autophagy of granulosa cells. AQP8 might be a potential target for diseases related to ovarian insufficiency.

## Highlight

AQP8-mediated H_2_O_2_ was involved in the autophagy of granulosa cells.
AQP8 deficiency could impair the elevation of H_2_O_2_ concentration through P-Tyr activation.
AQP8-mediated extracellular H_2_O_2_ may promote follicular atresia.

## Introduction

Aquaporin-8 (AQP8), as a transport facilitator, can control cellular oxidative stress by regulating H_2_O_2_ levels (Marchissio et al., 2012; Bertolotti et al., 2013). H_2_O_2_ is a reactive oxygen species (ROS) that serves as a secondary messenger in various signal transduction pathways. ROS and antioxidant deficiencies are involved in ovarian aging (Qian et al., 2016). Although high ROS levels are cytotoxic, low ROS levels are essential for cell physiology and survival. H_2_O_2_ transiently modulates tyrosine phosphatases and kinases and inhibits phosphatases to activate certain kinases (Finkel, 2011). H_2_O_2_ is generated *via* several approaches, such as oxidative phosphorylation in the mitochondria, oxidative protein folding in the endoplasmic reticulum, and NADPH oxidases in the plasma membrane (Kakihana et al., 2012). Some members of the aquaporin (AQP) family can transport H_2_O_2_ (Bienert et al., 2007; Miller et al., 2010). For instance, H_2_O_2_ can enter lymphoid cells through AQP8 and activate related growth factor signaling (Bertolotti et al., 2016). However, the mechanism by which AQP8 regulates the autophagy of ovary granulosa cells through H_2_O_2_ transport remains unclear.

Members of the AQP family can selectively and efficiently transport water molecules, and some of them transport small molecules, such as glycerol, urea, and H_2_O_2_, simultaneously. They are widely distributed in various animal tissues and serve important physiological functions. At present, few studies have concentrated on the roles of AQP in the reproductive system and various pathological processes (Sha et al., 2011; Zhang et al., 2012). Deletion of AQP3 affects sperm motility (Chen et al., 2011). Estrogen regulate the expression of AQP5 and AQP8 to abnormal embryo implantation (Zhang et al., 2015). A clinical study reported that the decreased uterine acceptance in patients with ovarian hyperstimulation is associated with decreased AQP2 expression in the endometrium (Zhang et al., 2016). And in yeast model, AQP8 can promote H_2_O_2_ transport (Bienert et al., 2007). Subsequently, studies have confirmed that AQP3 and AQP8 feature highly efficient H_2_O_2_ transport functions by systematically analyzing the H_2_O_2_ transport of various rat AQP family members overexpressed in HEK-293 cells (Miller et al., 2010). A recent study has shown that the migration of chemotactic T cells in skin contact hypersensitivity requires AQP3-mediated H_2_O_2_ transport (Hara-Chikuma et al., 2012). TNF stimulates the hyper H_2_O_2_ production of Nox2 on keratinized cell membranes, whereas the AQP3 synergistic transport of H_2_O_2_ regulating protein phosphatase 2A activates NF-KB signals to induce psoriasis (Hara-Chikuma et al., 2015). These results prove that some AQPs mediate the transport of H_2_O_2_ and regulate its harmful or beneficial functions.

We previously reported that *Aqp8* ^*−/−*^ mice have a more litter size (Su et al., 2010), with an increase in efficient follicles (Su et al., 2013). However, the mechanisms underlying these phenomena remain unclear. Previous studies have found that AQP8 is abundantly expressed in mouse (Su et al., 2010), rat (McConnell et al., 2002) and human (Li et al., 2013) ovaries. Thus, in this study, using *Aqp8* ^*−/−*^ mice and the follicular atresia model, we measured the intracellular H_2_O_2_ concentration and *Aqp8* expression level in granulosa cells and investigated the role of AQP8-transported H_2_O_2_ in the autophagy of granulosa cells.

## Material and methods

### Animal experiment

*Aqp8* ^−/−^ mice (C57BL/6 genetic background) were generated by targeted gene disruption (Yang et al., 2005). For this experiment, 5–6-week-old female mice were used. Mice were allowed free access to water and food in 12 h light and 12 h dark conditions. All animal experiments were reviewed and approved by the Committee on the Ethics of Animal Research of Dalian Medical University.

### Follicular atresia mouse model

*Aqp8* ^*+/+*^ and *Aqp8* ^−/−^ were intraperitoneally injected with 0.1 ml pregnant mare serum gonadotropin (PMSG) (1000 IU in 10 ml 0.9% NaCl, Ningbo a second hormone factory) or vehicle control (0.9% NaCl, 0.1 ml) at 16:00 into the mice. Mice were killed by cervical dislocation 0, 1, 2, 3, 4, and 5 days after PMSG treatment, and the ovary samples were excised. The ovaries were used for the collection of granulosa cells.

### Mouse primary granulosa cell collection and culture

Ovaries were excised from mice and placed in DMEM/F12 (GIBCO-BRL, 11039021) that was supplemented with 10% fetal bovine serum (GIBCO-BRL), 10 mg/ml of streptomycin sulfate (Sigma), and 75 mg/ml of penicillin G (Sigma). Granulosa cells were harvested by follicle puncture using a 25-gauge needle. After follicle puncture, granulosa cells were suspended in the appropriate solution for immunoblotting or H_2_O_2_ concentration.

For *in vitro* culture of granulosa cells under serum-free conditions, ovaries were collected, and granulosa cells were collected by follicle puncture, as described previously. The cells were seeded in 24-well plates and were allowed to attach overnight. The next morning, the medium and unattached cells were removed and replaced with serum-free media. After 24 h, the granulosa cells were fixed for immunofluorescence.

### Live cell station imaging of autophagic vacuoles

The adenovirus expressing GFP-LC3B (Ad-GFP-LC3B, C3006, Beyotime) was transfected into GCs grown on coverslips. After 24 h, cells were rinsed using PBS and then exposed to 2 h of H_2_O_2_ incubation. The distribution and fluorescence emitted by GFP-LC3B puncta were then observed under a live cell station (GE). Experiments were repeated three times.

### Western blot analysis

Protein was isolated by the previous description (Huang et al., 2020a). The freshly isolated granulosa cells were lysed with ice-cold radioimmunoprecipitation assay (RIPA) buffer that was supplemented with a protease inhibitor PMSF (Beyotime). To facilitate the complete solubilization of the cellular proteins, the cell lysates were incubated on ice for 30 min and then centrifuged (13000 g at 4°C for 30 min). The protein concentration was tested by using a BCA Protein Assay Kit. The whole-cell lysates (20 mg/lane) were separated by sodium dodecyl sulfate (SDS)–polyacrylamide gel electrophoresis and transferred to a polyvinylidene difluoride (PVFD) membrane (Millipore). After the nonspecific binding sites were blocked with 5% skim milk, the membrane was treated with the anti-LC3 rabbit polyclonal antibody (1:2000, Novus Biologicals), cleaved caspase-3 rabbit polyclonal antibody (diluted 1:1000, Cell Signaling Technology), and β-actin (1:1000, Beyotime) overnight at 4°C. The primary antibodies for Akt, pAkt-Ser473, Bax, Bcl-2, and P-Tyr were obtained from Cell Signaling Technology (Beverly, MA). The primary antibodies for Atg3 were obtained from Proteintech (Wuhan, China), while Beclin-1 was obtained from Santa Cruz Biotechnology (Santa Cruz, CA, United States). The immunoreactive bands were demonstrated by incubation with horseradish peroxidase (HRP)-conjugated goat anti-rabbit IgG (1:10000, Zhongshan Biotechnology) at room temperature for 1 h. The peroxidase activity was visualized with the enhanced chemiluminescence detection system (WBKLS500, Millipore). Integrated optical intensities of the immunoreactive protein bands were detected by using the DNR bioimaging system MicroChemi 4.2 and the quantified analysis by ImageJ software, normalized to *β-actin* values.

### Transmission electron microscopy test

To identify autophagic vacuoles at the ultrastructural level, the ovary was fixed with 2.5% glutaraldehyde in 0.1 M cacodylate buffer (pH 7.4) for more than 24 h at 4°C, rinsed in cacodylate buffer, postfixed in 1% OsO_4_ in cacodylate buffer, dehydrated, and embedded in Epon. Ultrathin sections were briefly contrasted with uranyl acetate and photographed with a transmission electron microscope (Hitachi 7100 Japan).

### Measurement of the intracellular hydrogen peroxide level

Intracellular hydrogen peroxide was measured by using the Hydrogen Peroxide Assay Kit (Beyotime, Nanjing, China). Briefly, 100–200 μl of hydrogen peroxide was added to detect the ratio of lysate to lysate. One million granulosa cells were treated with 100–200 μl of the hydrogen peroxide lysate, followed by sufficient homogenization to break and lyse the cells. It was then centrifuged at 12000 g at 4°C for 3–5 min, and the supernatant was collected. Then, 50 µl of the sample or standard was treated with 100 µl of the hydrogen peroxide detection reagent at room temperature (15–30°C). And then, it was immediately measured at A560 nm.

To evaluate intracellular H_2_O_2_ levels, 1×10^6^ cells/ml were washed twice in HBSS and incubated with 20 µM 2′,7′-dichlorofluorescein diacetate (DCFH-DA) (sigma). Granulosa cells were incubated for 30 min at 37°C. DCFH-DA was a small nonpolar and nonfluorescent molecule that diffuses into the cells, where it is enzymatically deacetylated by intracellular esterases to a polar nonfluorescent compound, which is oxidized to the highly green fluorescent 2,7-dichlorofluorescein (DCF). DCF fluorescence was measured using a multiwell plate reader (Synergy Neo HTS multimode microplate reader, BioTek) at excitation and emission wavelengths of 485 and 535 nm, respectively.

### RNA extraction and real-time PCR assay

Total RNA was isolated by the previous description (Huang et al., 2020b) using the TRIzol reagent (10296010, Invitrogen) and quantified using a NanoVueTM Plus Spectrophotometer (GE Healthcare, Buckinghamshire, United Kingdom). The A260/A280 ratio of the optical density was measured using the NanoVueTM Plus Spectrophotometer. The ratio was between 1.9 and 2.1 for all samples, indicating a good quality of RNA purity and yield. cDNA was synthesized from 2 µg of total RNA using the RT reagent Kit with gDNA Eraser (TaKaRa, RR047A). The qPCR solution contained 1 µl of cDNA, 1 µl of specific primers, and 10 µl of a 2X PCR SuperMix (AS111, TransGen Biotech) in a final volume of 20 µl. All primers were produced by lifespan (Milan, Germany) and listed as follows in the 5′-3′ direction: *Aqp8-F: ACA​CCA​ATG​TGT​AGT​ATG​GAC​CT*; *Aqp8-R: TGA​CCG​ATA​GAC​ATC​CGA​TGA​AG*; *β-actin-F: TGG​AAT​CCT​GTG​GCA​TCC​ATG​AAA​C*; *β-actin-R: TAA​AAC​GCA​GCT​CAG​TAA​CAG​TCC​G.*

The reaction conditions were as follows: polymerase activation and DNA denaturation (one cycle at 95°C for 30 s); denaturation, annealing, and extension (40 cycles at 95°C for 10 s and 60°C for 30 s); melting curve (65°C, with the temperature, gradually increased 0.5°C up to 95°C). mRNA expression was normalized to the level of *β-actin* mRNA. Changes in mRNA expression were calculated according to the 2^−△△Ct^ method. The amplification of β-actin mRNA was utilized to normalize the data.

### Analysis of transcriptome sequencing datasets

Raw transcriptome sequencing data were stored in FASTQ document format by Bcl2fastq (v2.17.1.14). Sequencing data quality was assessed by FastQC (v0.10.1). The raw data were preprocessed, the low-quality data were filtered, and the contamination and joint sequences were removed by cutadapt (version 1.9.1). Then, gene expressions were calculated by HTSeq (V 0.6.1) with FPKM (Fragments per Kilobase per Million reads) (Mortazavi et al., 2008). Differentially expressed genes (DEGs) in the ovary (group: control and PMSG with treatment for 2 days or 4 days) were analyzed by using the R package edgeR (v3.4.6) using |fold change|>2 and a *p*-value < 0.05.

### Statistical analysis

Statistical analysis was performed by analysis of variance (ANOVA). Significant differences between treatment groups were determined by Duncan’s multiple range tests. *p* < 0.05 was considered statistically significant. For statistical comparisons between two groups, an independent-sample *t*-test was used.

## Results

### Autophagy and apoptosis decrease in *Aqp8* ^*−/−*^ mouse follicular granulosa cells

**FIGURE 1:**
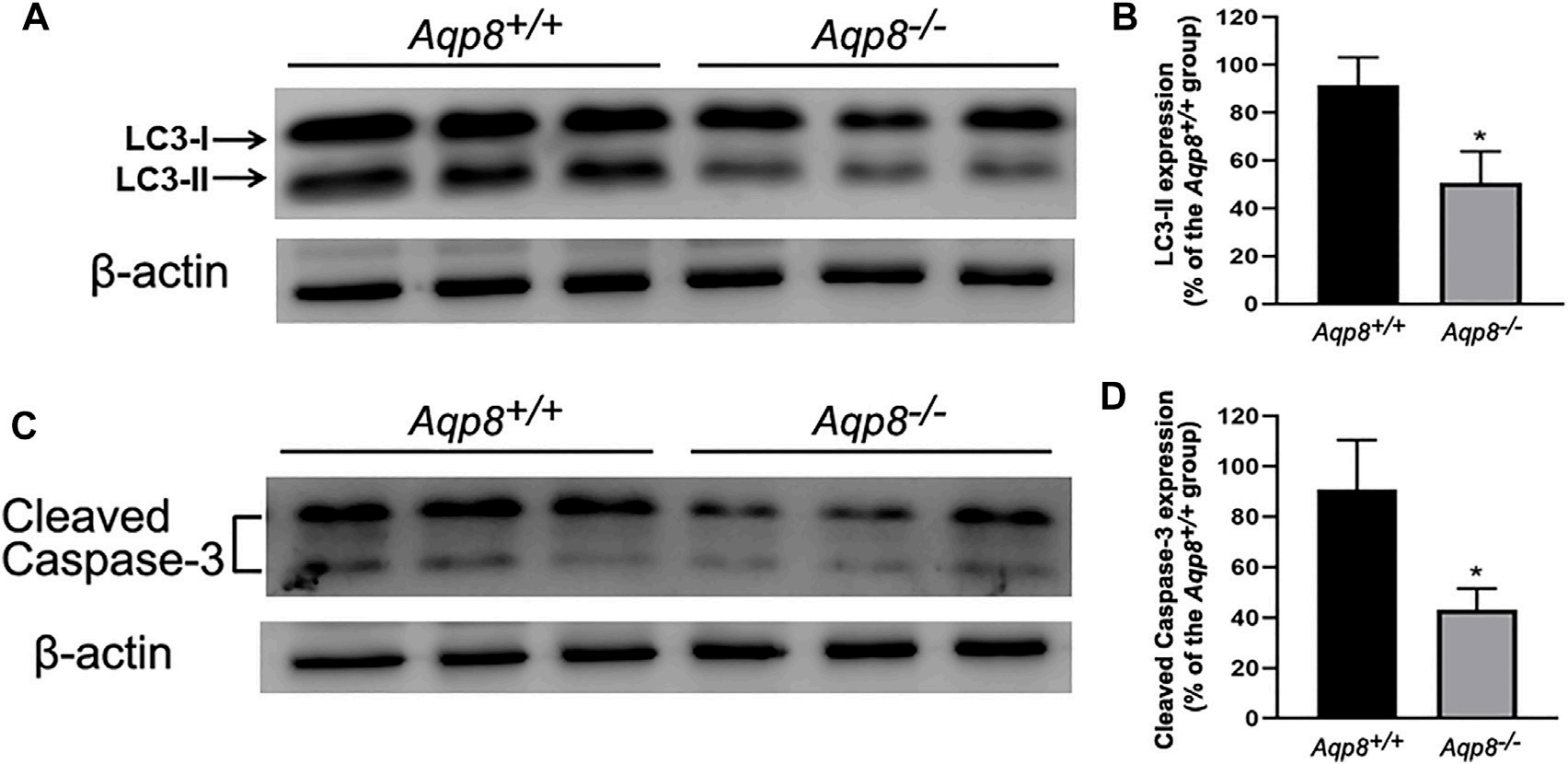
Autophagy and apoptosis decrease in *Aqp8* ^−/−^ mouse follicular granulosa cells. The expressions of cleaved caspase-3 and LC3-II proteins in granulosa cells from *Aqp8* ^*+/+*^ and *Aqp8* ^*−/−*^ mice. Densitometric quantification and representative immunoblots of LC3 proteins **(A,B)** or cleaved caspase-3 **(C,D)**. Experiments were repeated three times, and data were expressed as mean ± SD. *p <0.05 were considered statistically significant.

Microtubule-associated light-chain protein 3 (LC3) is an autophagy marker used to evaluate granulosa cell autophagy. Granulosa cells freshly isolated from *Aqp8* ^*+/+*^ and *Aqp8* ^*−/−*^ mice were used. The results of Western blot analysis using specific antibodies against the autophagy marker LC3 and apoptosis marker cleaved caspase-3 were shown in Figure 1. Densitometric analysis normalized by the β-actin content indicated that the protein contents of LC3 and cleaved caspase-3 significantly decreased compared with the control, suggesting that AQP8 was involved in the development of follicular granulosa cells *via* autophagy and apoptosis.

### Localization of autophagosomes in granulosa cells

**FIGURE 2:**
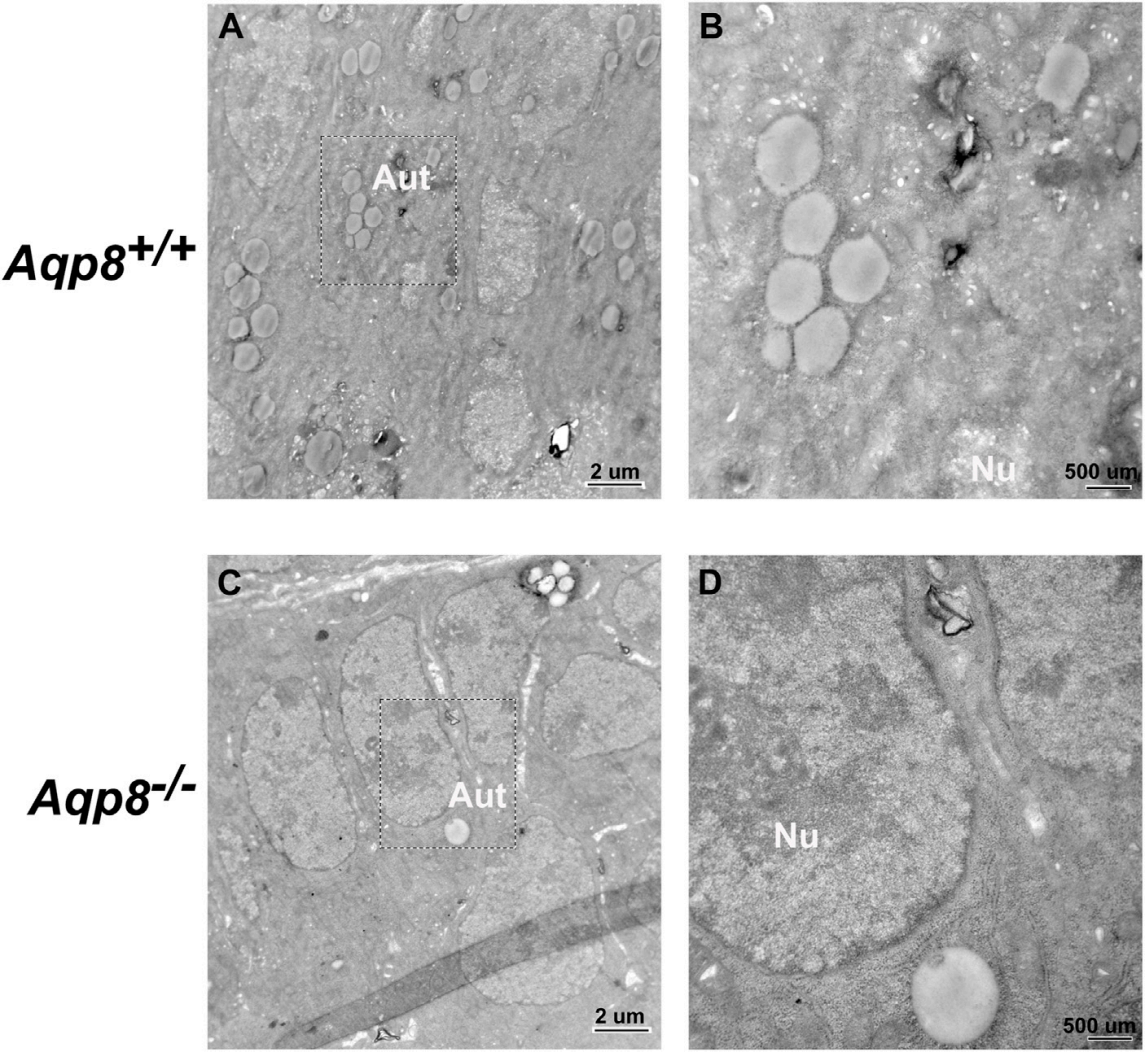
Transmission electron microscopic images of granulosa cells in *Aqp8* ^+/+^ and *Aqp8* ^−/−^ mice. Representative images from transmission electron microscopy of granulosa cells from *Aqp8* ^*+/+*^ **(A,B)** and *Aqp8* ^*−/−*^ **(C,D)** mice. High-magnification images indicated nucleus (Nu) and autophagosomes (Aut). The scale was indicated on the lower right corner of each image.

Transmission electron micrographs showed that autophagosomes existed in the follicular granulosa cells of *Aqp8* ^*+/+*^ and *Aqp8* ^*−/−*^ mice. Fewer autophagosomes localized in the granulosa cells of the *Aqp8* ^*−/−*^ mice than in those of the *Aqp8* ^*+/+*^ mice (Figure 2).

### Aquaporin-8 channel mediates H_2_O_2_ uptake and AQP8-dependent H_2_O_2_ permeability in granulosa cells

**FIGURE 3:**
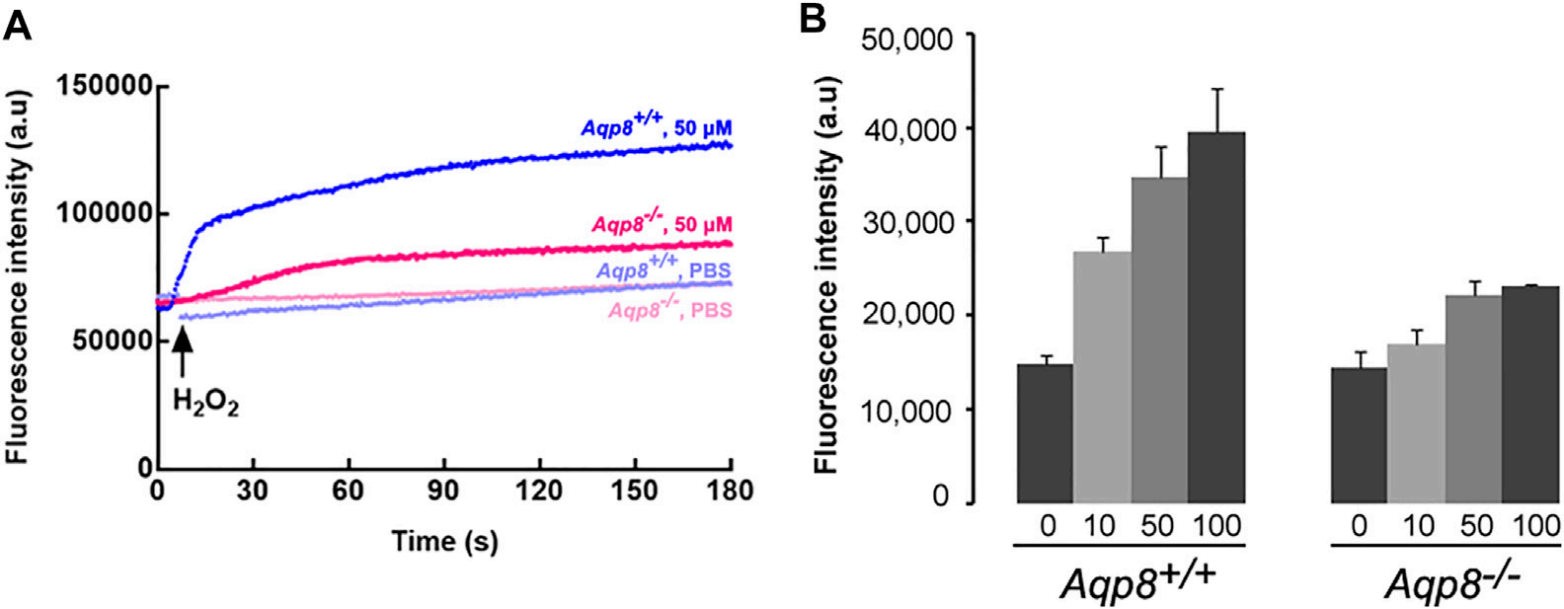
H_2_O_2_ uptake into primarily cultured granulosa cells. **(A)** Representative fluorescence intensity of CM-H2DCFDA. **(B)** Granulosa cells were incubated with H_2_O_2_ (0–100 mM), and cellular H_2_O_2_ was detected by CM-H2DCFDA fluorescence using a plate reader. Increased fluorescence intensity could be detected at 15 s after treatment with H_2_O_2_. Data were shown as mean ± SD.

Considering that AQP8 is expressed on granulosa cells (Su et al., 2010), we used *Aqp8* ^*−/−*^ mice to determine whether or not AQP8 is required for the efficient entry of exogenous H_2_O_2_. To investigate H_2_O_2_ transport across the plasma membrane, we incubated DFCH-DA into granulosa cells. The addition of PBS or 50 µM H_2_O_2_ clearly activated DFCH-DA to DCF, as determined by 488/510 nm shifts on a microplate reader (Figure 3A). The aforementioned experiments provided additional evidence that H_2_O_2_ cannot freely permeate through the plasma membrane and identified AQP8 as an efficient transporter. Clearly, H_2_O_2_ import was severely impaired with AQP8 silencing.

Meanwhile, we investigated whether or not AQP8 can transport extracellular H_2_O_2_ into granulosa cells. Intracellular H_2_O_2_ was measured in primary granulosa cell cultures from *Aqp8* ^*+/+*^ and *Aqp8* ^−/−^ mice after the extracellular addition of 10–100 μM H_2_O_2_ using the fluorescent dye CM-H2DCFDA, which reacted with ROS, including H_2_O_2_. Results showed that the cellular H_2_O_2_ level was significantly higher in the granulosa cells of the *Aqp8* ^*+/+*^ mice than that in those of the Aqp8^−/−^ mice (Figure 3B), indicating the involvement of AQP8 in H_2_O_2_ transport in granulosa cells. The ability of AQP8 to transport H_2_O_2_ across the plasma membrane was tested. Figure 3B showed that the basal intracellular H_2_O_2_ level was significantly affected by AQP8, revealing that the channel activity of this AQP isoform modulated the entry of physiologically produced H_2_O_2_ into the cells.

### Aquaporin-8 participates in granulosa cell autophagy during follicular development and atresia

**FIGURE 4:**
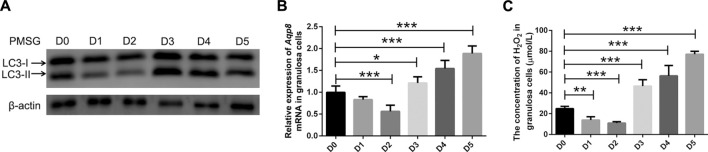
AQP8 involvement in granulosa cell autophagy during follicular development and atresia. **(A)** Expression of LC3-II proteins in granulosa cells from immature mice at different times (0–5 days) after pregnant mare serum gonadotropin injection (10 IU, intraperitoneally). **(B)** qRT-PCR was performed to measure the mRNA levels of *Aqp8* in GCs. The expression of *Aqp8* mRNA in granulosa cells from immature mice at different times (0–5 days) after pregnant mare serum gonadotropin injection. **(C)** Concentration of H_2_O_2_ in granulosa cells from immature *Aqp8* ^*+/+*^ mice at different times (0–5 days) after pregnant mare serum gonadotropin injection (10 IU, intraperitoneally). Values are presented as the means ± SD. *p < 0.05, **p < 0.01, and ***p < 0.001.

Autophagy, as an important process of programmed cell death, regulates follicular homeostasis in rats (Choi et al., 2010). Follicular development and atresia *in vivo* model was established in immature mice. Granulosa cell autophagy was determined by measuring the expression levels of LC3-II/LC3-I. As shown in Figure 4A, LC3-II expression in the granulosa cells was significantly downregulated 1 and 2 days after PMSG injection compared with the granulosa cells of immature mice without the treatment of exogenous gonadotropin (day 0). LC3-II expression was upregulated on day 3 and maintained until day 5. Furthermore, the expression of *Aqp8* was measured. After PMSG injection, the expression of *Aqp8* was significantly downregulated on days 1 and 2 and then increased on days 3, 4, and 5 (Figure 4B). Ovarian granulosa cells were isolated from each stage of the atresia model, and the concentration of H_2_O_2_ in granulosa cells was measured. The concentration of H_2_O_2_ in granulosa cells decreased during follicular development but increased during atresia (Figure 4C), suggesting that AQP8 mediated H_2_O_2_ transport and autophagy in granulosa cells to regulate follicular development and atresia.

### Aquaporin-8 inhibits pAkt and phosphorylated tyrosine signaling

**FIGURE 5:**
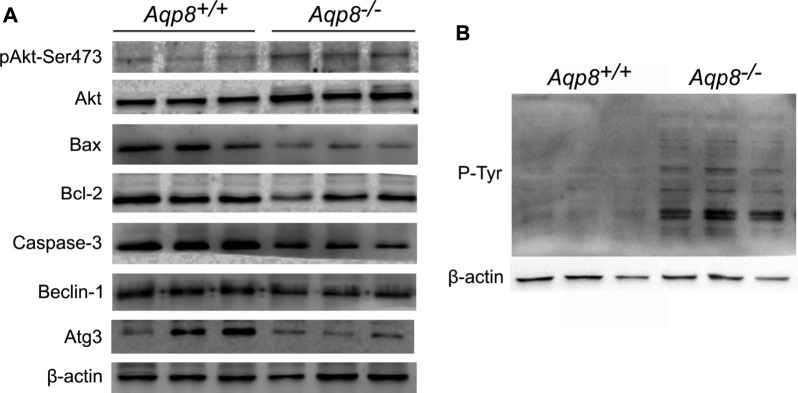
AQP8 inhibits pAkt and phosphorylated tyrosine (P-Tyr) signaling. **(A)** Western blot detection of the relevant signaling pathway. **(B)** Western blot of P-Tyr, which could be affected by H_2_O_2_.

The mechanism by which AQP8 regulates the autophagy of granulosa cells was further explored. Results showed that AQP8 can facilitate the absorption of H_2_O_2_ into granulosa cells and mediate downstream intracellular signaling. As shown in Figure 5A, the phosphorylation level of Akt in the granular cells of the *Aqp8* ^*−/−*^ mice was increased. The expression levels of proteins related to apoptosis (Bax and caspase-3) and autophagy (Beclin-1 and Atg-3) decreased in the granulosa cells of the *Aqp8* ^*−/−*^ mice. Meanwhile, phosphorylated tyrosine signaling was activated in the granulosa cells of the *Aqp8* ^*−/−*^ mice (Figure 5B). And the absorption of H_2_O_2_ in the granulosa cells of the *Aqp8* ^*+/+*^ mice inhibited the PI3K signaling pathway and promoted granulosa cell death and autophagy.

### AQP8 mediated H_2_O_2_ uptake affects granulosa intracellular autophagy

**FIGURE 6:**
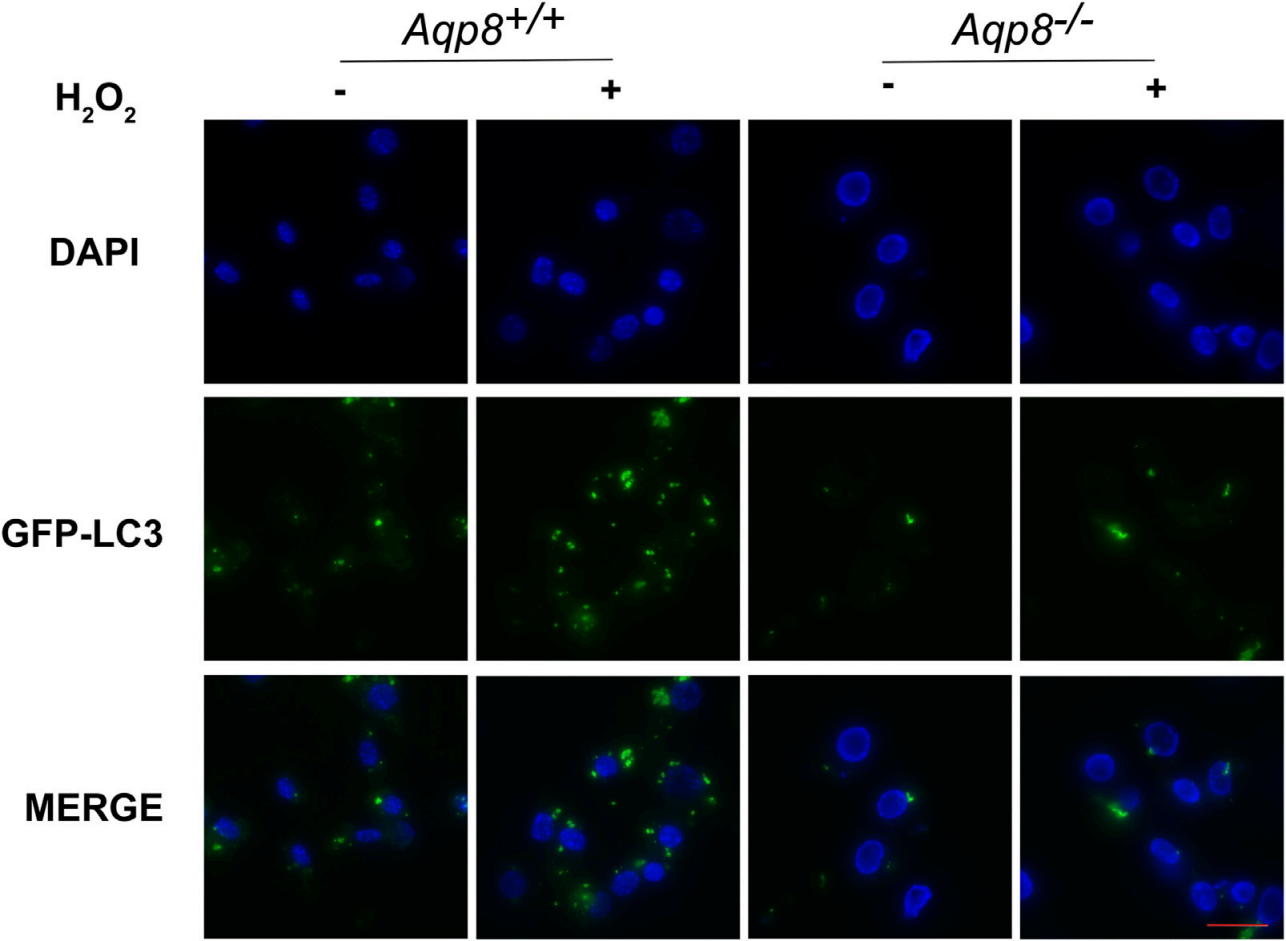
AQP8-mediated H_2_O_2_ uptake influences granulosa intracellular autophagy. GCs transfected with the GFP-LC3B plasmid for 24 h were incubated with 200 μM H_2_O_2_ for 1 h and rinsed in PBS. A live cell station was employed to observe the GFP fluorescent puncta in GCs. The nuclei were counterstained with DAPI (blue). Scale bar = 10 μm.

We next investigated whether or not AQP8 influences autophagy by oxidative stress. An *in vitro* culture experiment revealed that AQP8 was involved in granulosa cell autophagy through the transport of H_2_O_2_. Primary cultured GCs were transfected with the GFP-LC3B plasmid, incubated with or without 200 μM H_2_O_2_ for 2 h, and then rinsed with PBS. Immunofluorescence images of granulosa cells were obtained in a live cell station. As shown in Figure 6, AQP8 obviously induced autophagosome formation in the cells with H_2_O_2_ exposure, displaying that AQP8 facilitated intracellular autophagy of granulosa cells through H_2_O_2_ absorption.

### Integrated analysis of the ovary transcriptome in mice with PMSG treatment

**FIGURE 7:**
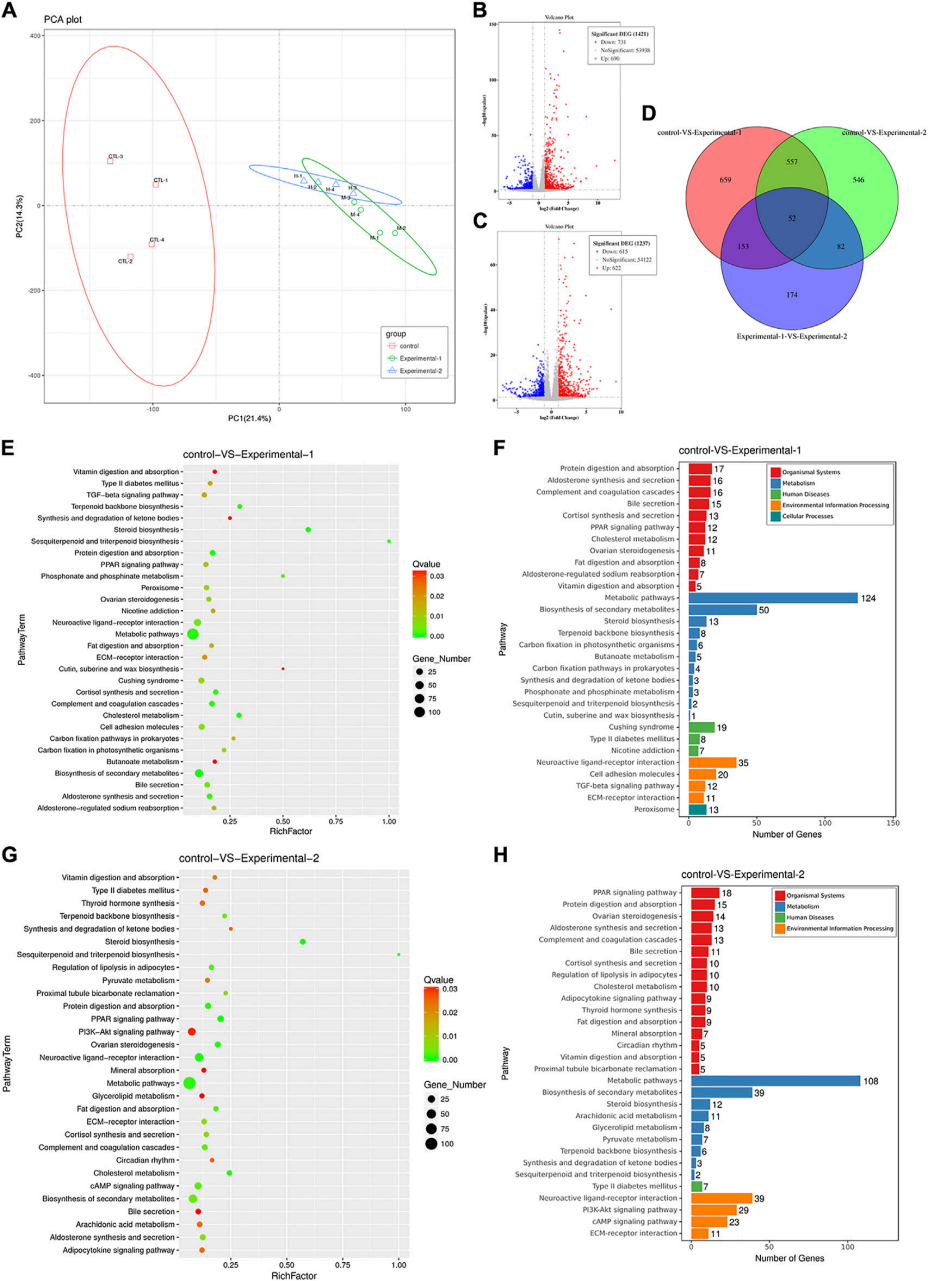
Analysis of the ovary transcriptome level after PMSG treatment. **(A)** Principal component analysis (PCA) was performed on the ovary (control, PMSG treatment for 2 days or 4 days), and the R function prcomp was used to analyze the samples at the transcriptome level. The volcano plot shows differentially expressed genes of three groups in the ovary with PMSG treatment for 2 days **(B)** and 4 days **(C)**. The red circle represents genes that are upregulated in gene expression, the blue circle represents genes that are downregulated in gene expression, and the gray circle represents insignificant genes. **(D)** Venn plot shows the correlation of differential genes expressed among three groups. The bubble plot represented the enriched KEGG pathways of differentially expressed genes in the ovary with PMSG treatment for 2 days **(E,F)** and 4 days **(G,H)**. The red color indicated upregulated pathways, and the blue color indicated downregulated pathways. The bubble size represents the number of genes associated with pathways in each group, and the connected color indicates the *p*-value across the connected groups. Control, treatment with saline; experiment 1, treatment with PMSG for 2 days; experiment 2, treatment with PMSG for 4 days.

Transcriptome analysis of the ovary was performed in mice with PMSG treatment for 2 days or 4 days. Principal components, differentially expressed genes (DEGs), and KEGG pathways were analyzed. The principal components of each group between parents and offspring showed obvious differences (Figure 7A). To further study the mechanisms underlying follicular development and atresia, we analyzed the DEGs and KEGG pathways between different groups. In total, 1,421 DEGs with |fold change|> 2 and *p*-value < 0.05 were annotated in the ovary (groups: control and injected with PMSG for 2 days), of which 690 genes were upregulated and 731 genes were downregulated (Figure 7B). In total, 1,237 DEGs with |fold change| > 2 and *p* value < 0.05 were annotated in the ovary (groups: control and injected with PMSG for 4 days), of which 622 genes were upregulated and 615 genes were downregulated (Figure 7C). In addition, DEG analyses of the three cohorts (control, injected with PMSG for 2 days or 4 days) overlapped, with 52 common DEGs obtained (Figure 7D). KEGG pathway enrichment analysis was also performed to study the pathway with significant DEG enrichment. Functional enrichment results revealed that 30 KEGG pathways of DEGs in the ovaries of the mice injected with PMSG for 2 days or 4 days were statistically significantly enriched, respectively (Figures 7E,G). The KEGG pathways in the control mice and mice injected with PMSG for 2 days included five categories: organismal systems, metabolism, human diseases, environmental information processing, and cellular processes in the ovary (Figure 7F). The KEGG pathways in the control mice and mice injected with PMSG for 4 days included four categories: organismal systems, metabolism, human diseases, and environmental information processing in the ovary (Figure 7H). Organismal systems and metabolism are the most enriched pathways, suggesting that they might play important roles in follicular development and atresia.

## Discussion

In the present study, we demonstrated that AQP8 induced autophagy by transporting H_2_O_2_ into granulosa cells during follicular atresia. During follicular growth and development, only less than 1% of oocytes sealed and protected by granular cells can form dominant follicles and be discharged from the body, whereas the remaining 99% of the follicles are degenerated, leading to atresia. Many studies have found that mammalian follicular development and atresia are primarily controlled by granulosa programmed cell death. In addition, at least five types of death ligands involved in programmed cell death induce particles, including TNF-alpha, Fas, TRAIL, APO-3, and PEG-5 ligands and their receptors (Manabe et al., 2004; Hurst et al., 2006; Lin and Rui, 2010). However, the follicle selection process control of the molecular mechanism of granular cell death remains unclear.

During follicular development, granulosa cell apoptosis is not the only mechanism to control follicular atresia. Autophagy and cell necrosis have been observed in the follicles of other animals (Kovacs et al., 1992; D'Herde et al., 1996; Shao et al., 2022). In recent years, some studies have found that granulosa autophagy is also involved in follicular atresia (Choi et al., 2010; Choi et al., 2011; Matsuda et al., 2012; Zhou et al., 2019).Gonadotropin could reduce the granulosa cell autophagy by activating the PI3K/Akt signaling pathway with inhibition of mTOR (Choi et al., 2014), The expression of Beclin-1 and LC3 would increase in the ovary through the oxidative stress of mitochondrial damage after treatment with harmful chemical substances in cigarette (Gannon et al., 2013; Furlong et al., 2015). Conversely, inhibiting the expressions of Akt and mTOR proteins activate the AMPK pathway, which promotes granulosa autophagy and decreases the number of original follicles affecting reproduction (Shen et al., 2017; Shen et al., 2018). Exogenous addition of oxidizing LDL can trigger autophagy in human granulosa cells (Duerrschmidt et al., 2006). Furthermore, the circadian clock system is also involved in the granulosa cell autophagy through the regulation of nuclear receptor subfamily 1 group D member 1 (NR1D1) (Zhang et al., 2022). These findings indicate that autophagy is directly involved in the regulation of granulosa death during follicular development, whereas oxidative stress may play an important role in the regulation of granulosa autophagy.

The ROS content in mammalian cells is strictly controlled. ROS are the central elements of cell proliferation, apoptosis and other signal pathway. The H_2_O_2_ concentration decreases in the follicular fluids of animals, such as bovine (Gupta et al., 2011) and swine (Basini et al., 2008), with the follicular development, whereas the upper limit of the ROS concentration in human follicular fluids is strictly controlled (Jana et al., 2010). The balance between ROS and antioxidants in follicles plays an important role in follicular development. The physiological levels of ROS and antioxidants jointly regulate the follicular formation, and the continuous action or increase in the ROS concentration interferes with the intracellular redox reaction and leads to oxidative stress (Agarwal et al., 2008). Recent findings have shown that ROS exerts cytotoxic effects because of oxidative damage, and H_2_O_2_ activates apoptotic proteases through oxidative pressure or destroys cytochromes released by intracellular mitochondria to induce the apoptosis of Jurkat T lymphocytes (Hampton and Orrenius, 1997), which has the same effect on bovine follicular granulosa cells (Hennet et al., 2013). However, increasing evidence suggests that ROS, especially H_2_O_2_, can activate or inactivate multiple signaling pathways as signaling molecules by activating or inactivating phosphorylated proteins in cells (Rhee, 2006; Forman et al., 2010).

H_2_O_2_ is a relatively stable class of reactive oxygen molecules *in vivo*. H_2_O_2_ produced by NADPH oxidase on the cell surface can act as a secondary messenger responding to extracellular stimuli, such as growth factors, hormones, and cytokines (Bae et al., 1997; Rhee, 2006; Schroder and Eaton, 2008). As a signaling molecule, H_2_O_2_ activates ERK1/2 and the PI3K-Akt signaling pathway corresponding to the inhibition of PTP1B and PTEN pathways, whereas ERK and PTEN signaling pathways are crucial for ovulation (Blanc et al., 2003; Tonks, 2005). Therefore, the H_2_O_2_ concentration should be controlled and maintained to ensure its role in signal transduction. The concentration of H_2_O_2_ in non-atretic follicular fluids is higher than that in atretic follicles, suggesting that ROS in follicular fluids could regulate follicular atresia (Hennet et al., 2013). Exogenous H_2_O_2_ can activate the JNK signaling pathway and induce autophagy in mesenchymal stem cells (Liu et al., 2015). Intracellular H_2_O_2_ levels rapidly reach a certain threshold to play their molecular signaling function (Rhee, 2006). Extracellular H_2_O_2_ can enter cells through free diffusion, but free diffusion can not instantly increase the intracellular concentration of H_2_O_2_. Thus, the regulation of H_2_O_2_ transport possibly depends on selective membrane. The expression of Beclin-1 and LC3 would increase in the ovary through the oxidative stress of mitochondrial damage after treatment with harmful chemical substances in cigarette channel proteins (Rhee, 2006; Kruger et al., 2021).

The autophagy of granulosa cells increased during follicular atresia in the *Aqp8* ^*+/+*^ mice, whereas the autophagy and apoptosis of ovarian granulosa cells significantly decreased in the *Aqp8* ^*−/−*^ mice. Primary granulosa cells of the *Aqp8* ^*+/+*^ and *Aqp8* ^*−/−*^ mice were cultured *in vitro*, and different concentrations of H_2_O_2_ were added externally. The H_2_O_2_ concentration in the granulosa cells of the *Aqp8* ^*+/+*^ mice was significantly higher than that in the granulosa cells of the *Aqp8* ^*−/−*^ mice, indicating that AQP8 can mediate the efficient transport of H_2_O_2_ outside granulosa into cells. Further studies found that the H_2_O_2_ concentration in the granulosa cells increased during follicular atresia in mice. H_2_O_2_ transported by AQP8 can act as a secondary messenger to regulate intracellular signal transmission.

In conclusion, we provided a novel mechanism by which AQP8 transported extracellular H_2_O_2_ and induced cellular autophagy in granulosa cells, resulting in the increase of follicular atresia. This regulative mechanism may shed light on the treatment of ovarian insufficiency.

## Data Availability

The original contributions presented in the study are included in the article/Supplementary Material; further inquiries can be directed to the corresponding authors.

## Abbreviations

AQP, aquaporin; ROS, reactive oxygen species; H_2_O_2_, hydrogen peroxide; LC3, light-chain protein 3; PMSG, pregnant mare serum gonadotropin; PCR, polymerase chain reaction; Akt, protein kinase B; PCD, programmed cell death.
