# Supplementary material for: Aquaporin-8 transports hydrogen peroxide to regulate granulosa cell autophagy
Source: Front Cell Dev Biol. 2022 Aug 23;10:897666. doi: 10.3389/fcell.2022.897666 (PMC9445271; doi:10.3389/fcell.2022.897666)
Supplement: Supplementary file 1 [file Presentation1.PPTX]

## Slide 1
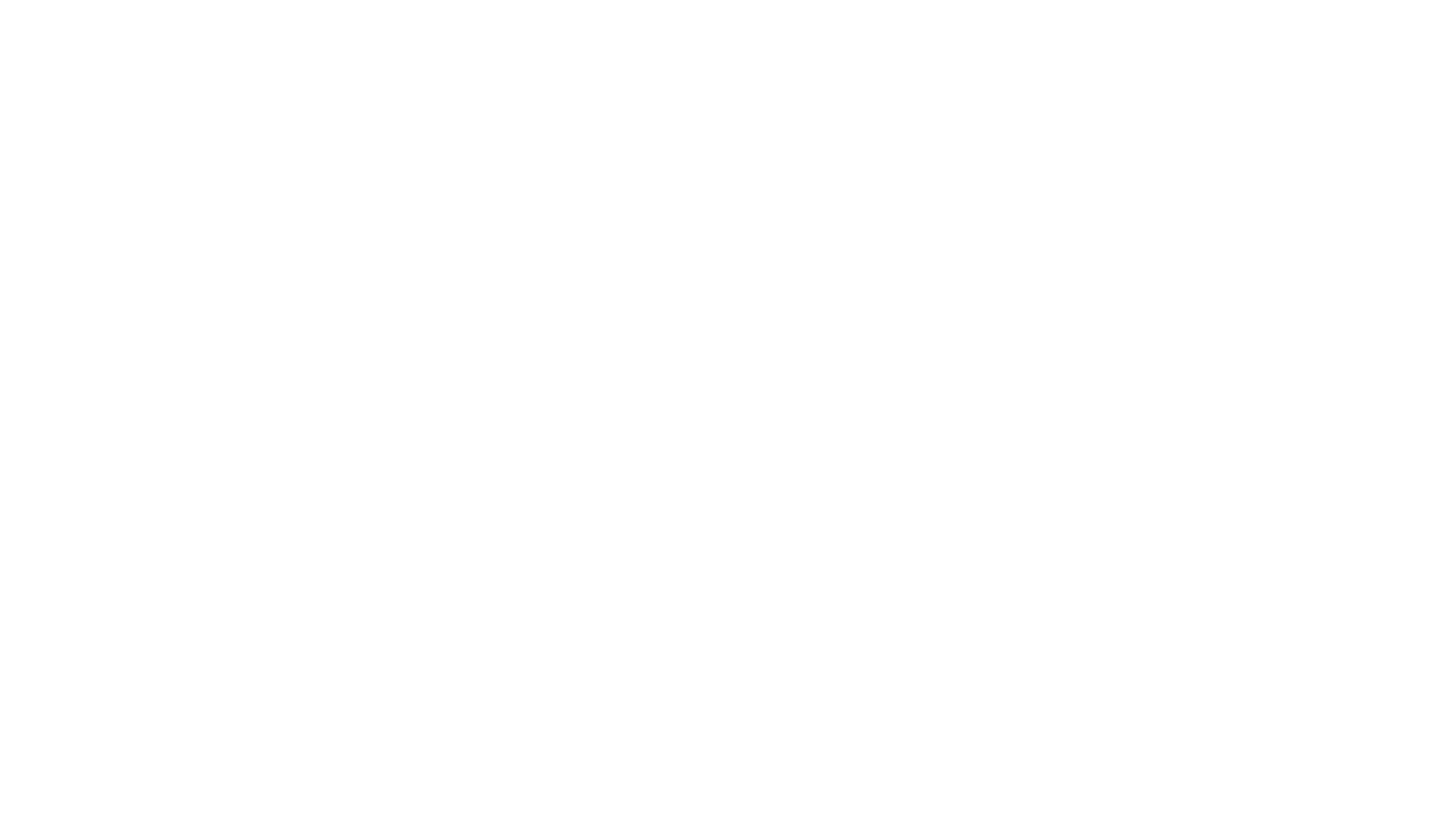

#

## Slide 2
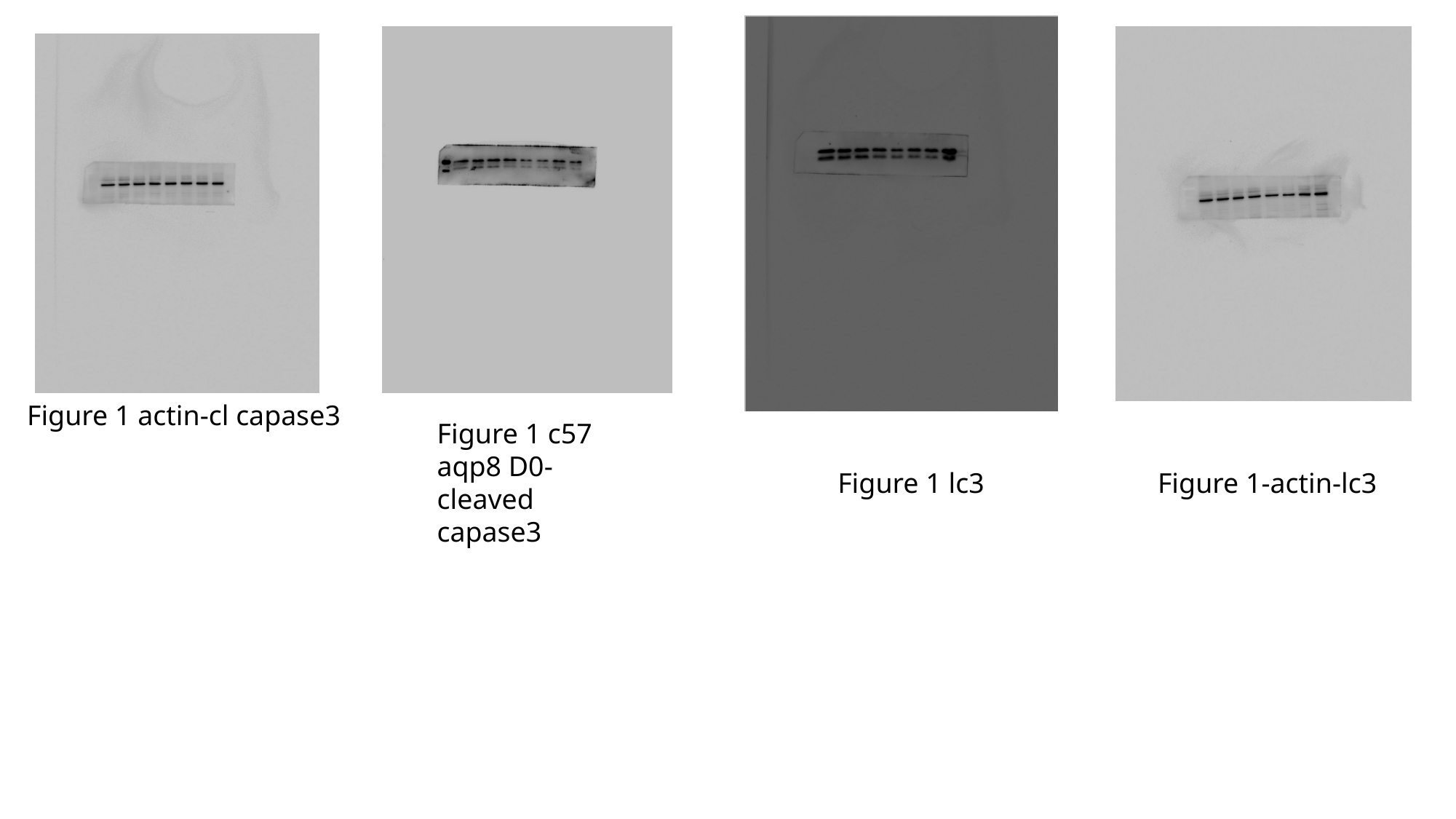

Figure 1 actin-cl capase3
Figure 1 c57 aqp8 D0-cleaved capase3
Figure 1 lc3
Figure 1-actin-lc3

## Slide 3
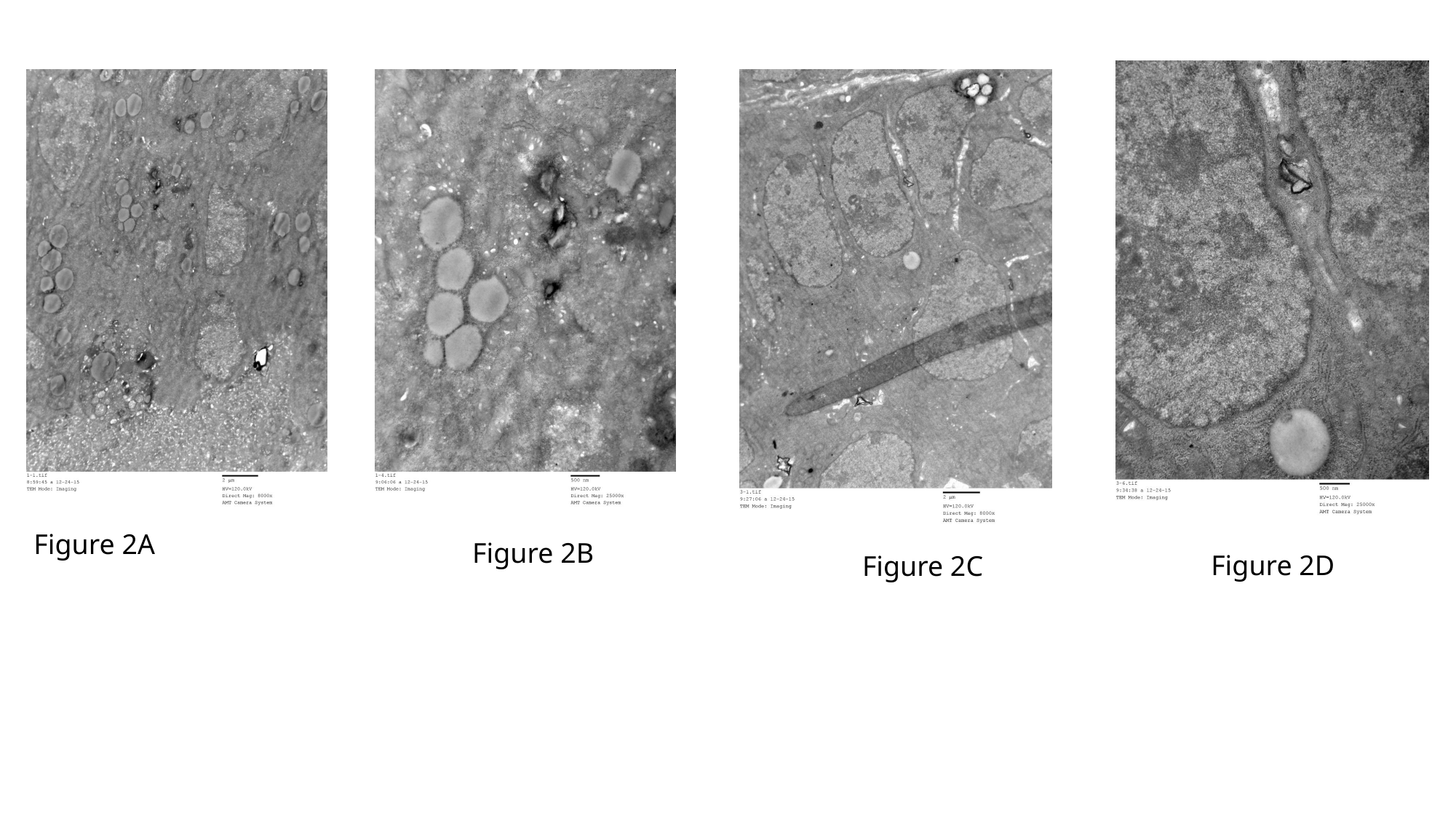

Figure 2A
Figure 2B
Figure 2D
Figure 2C

## Slide 4
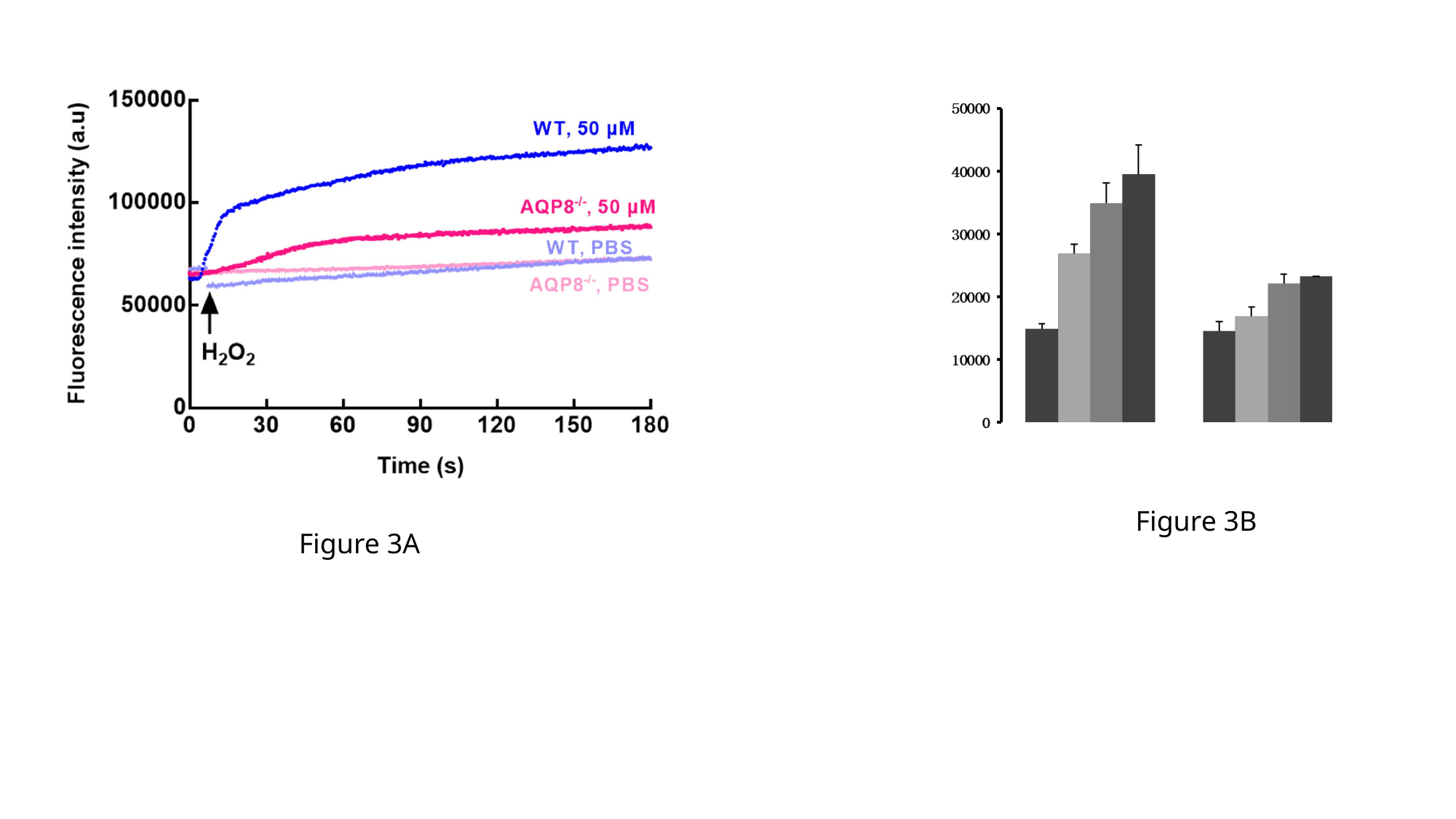

Figure 3B
Figure 3A

## Slide 5
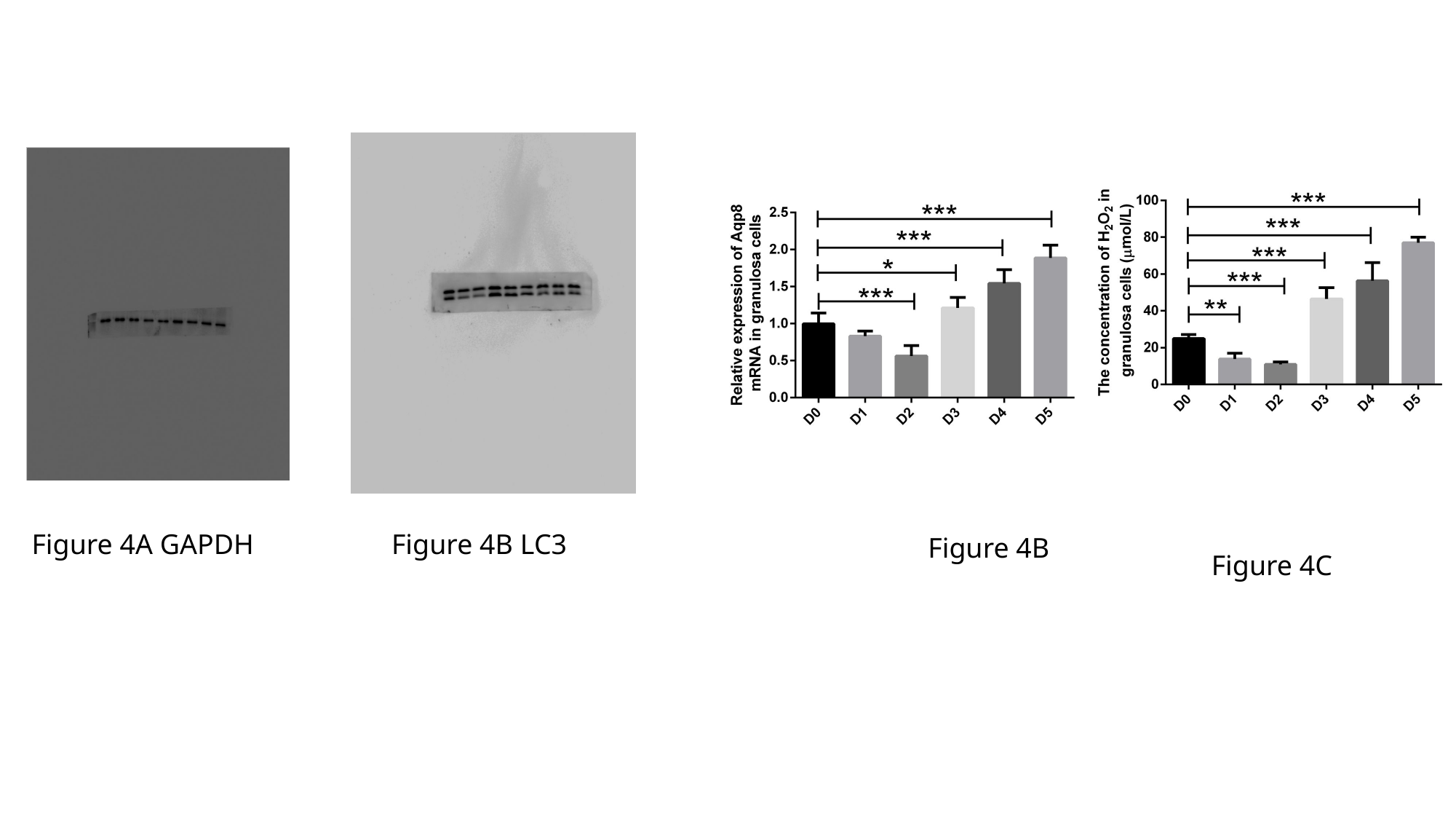

Figure 4A GAPDH
Figure 4B LC3
Figure 4B
Figure 4C

## Slide 6
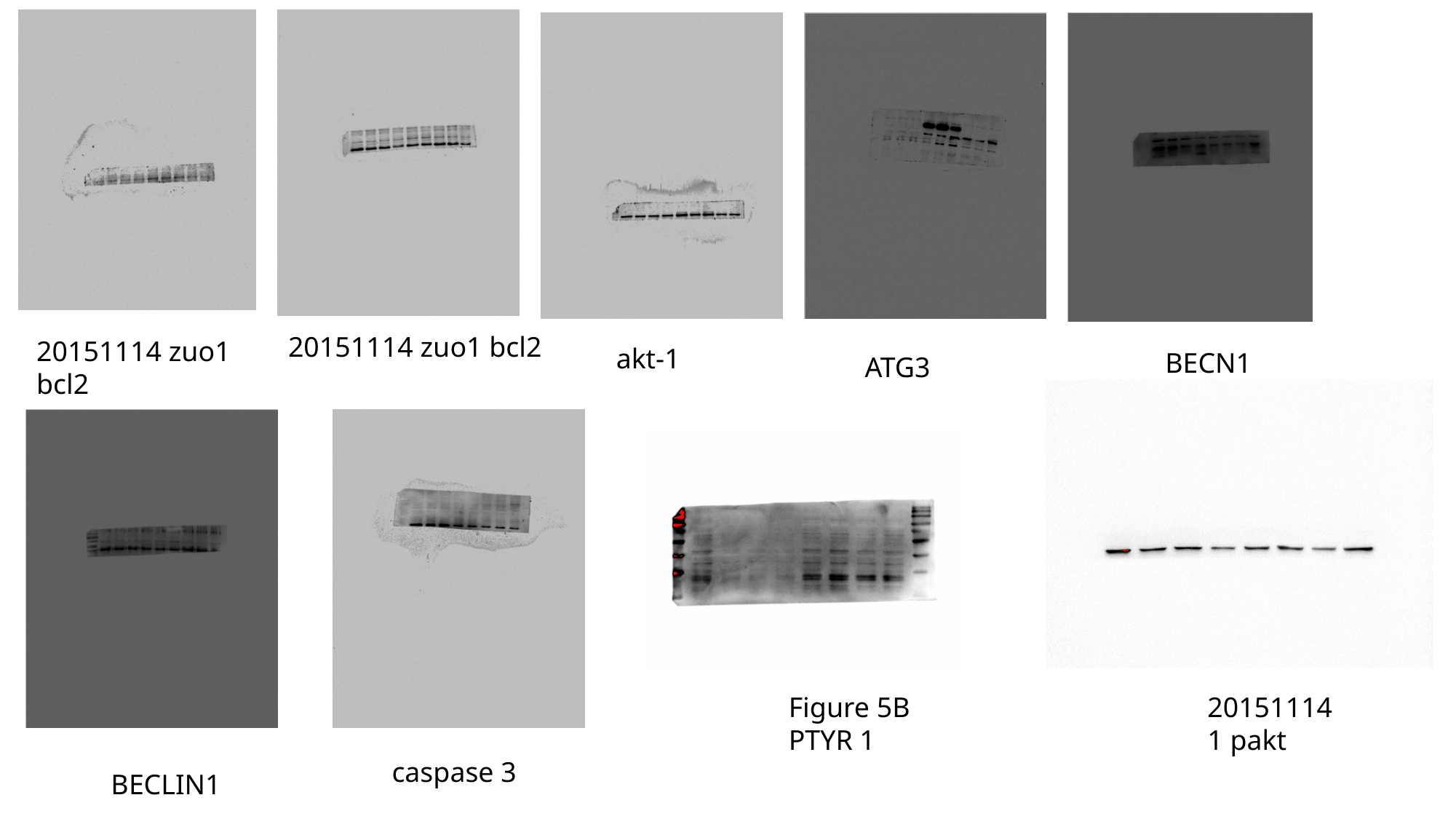

20151114 zuo1 bcl2
20151114 zuo1 bcl2
akt-1
BECN1
ATG3
Figure 5B PTYR 1
20151114 1 pakt
caspase 3
BECLIN1
